# The inference of gray whale (Eschrichtius robustus) historical population attributes from whole-genome sequences

**DOI:** 10.1186/s12862-018-1204-3

**Published:** 2018-06-07

**Authors:** Anna Brüniche-Olsen, Rick Westerman, Zuzanna Kazmierczyk, Vladimir V. Vertyankin, Celine Godard-Codding, John W. Bickham, J. Andrew DeWoody

**Affiliations:** 1 Department of Forestry & Natural Resources, Purdue University, West Lafayette, IN 47905 USA; 2 Department of Horticulture & Landscape Architecture, Purdue University, West Lafayette, IN 47907 USA; 3 School of Forensic and Applied Sciences, University of Central Lancashire Preston, Preston, PR1 2HE UK; 4 Kronotsky State Nature Biosphere Reserve, Elizovo, Kamchatka 684010 Russia; 5 The Institute of Environmental and Human Health, Department of Environmental Toxicology, Texas Tech University, 1207 Gilbert Drive, Lubbock, TX 79409 USA; 6 Department of Wildlife & Fisheries Sciences, Texas A&M University, College Station, TX 77843 USA

**Keywords:** Admixture, Relatedness, Runs-of-homozygosity, Cetacean, Demographic history

## Abstract

**Background:**

Commercial whaling caused extensive demographic declines in many great whale species, including gray whales that were extirpated from the Atlantic Ocean and dramatically reduced in the Pacific Ocean. The Eastern Pacific gray whale has recovered since the 1982 ban on commercial whaling, but the Western Pacific gray whale—once considered possibly extinct—consists of only about 200 individuals and is considered critically endangered by some international authorities. Herein, we use whole-genome sequencing to investigate the demographic history of gray whales from the Pacific and use environmental niche modelling to make predictions about future gene flow.

**Results:**

Our sequencing efforts and habitat niche modelling indicate that: i) western gray whale effective population sizes have declined since the last glacial maximum; ii) contemporary gray whale genomes, both eastern and western, harbor less autosomal nucleotide diversity than most other marine mammals and megafauna; iii) the extent of inbreeding, as measured by autozygosity, is greater in the Western Pacific than in the Eastern Pacific populations; and iv) future climate change is expected to open new migratory routes for gray whales.

**Conclusion:**

Our results indicate that gray whale genomes contain low nucleotide diversity and have been subject to both historical and recent inbreeding. Population sizes over the last million years likely peaked about 25,000 years before present and have declined since then. Our niche modelling suggests that novel migratory routes may develop within the next century and if so this could help retain overall genetic diversity, which is essential for adaption and successful recovery in light of global environmental change and past exploitation.

**Electronic supplementary material:**

The online version of this article (10.1186/s12862-018-1204-3) contains supplementary material, which is available to authorized users.

## Background

Widespread commercial whaling during the last two centuries unsustainably harvested many whale populations [1]. Whale products such as oil, meat, blubber, and ambergris were commercially important and overharvesting greatly diminished many whale populations [2–5]. In 1982 the International Whaling Commission (IWC) instituted a moratorium on commercial whaling (https://iwc.int/commercial), and although some whale populations have since recovered to near their pre-whaling abundance, others remain compromised. Recent, anthropogenic bottlenecks due to commercial whaling can be contrasted with more ancient, natural bottlenecks often associated with climate and/or ecological change [3].

Great whales are important for marine ecosystems, as they facilitate nutrient transfer in the water column and stabilize ecosystems by increasing biodiversity [6]. Whales are associated with areas of high primary productivity, and their sensitivity to environmental changes make them prime indicators of ecological perturbations [7]. The marine ecosystem is rapidly changing due to anthropogenic impacts [8–10], most of which have unknown consequences for the future of marine environments and marine mammals [11, 12]. Scientists are just beginning to understand how large marine mammals have responded to past climatic cycles [13–15], and models predict that range and distribution patterns will shift towards the poles in the face of global warming [16].

**Fig. 1:**
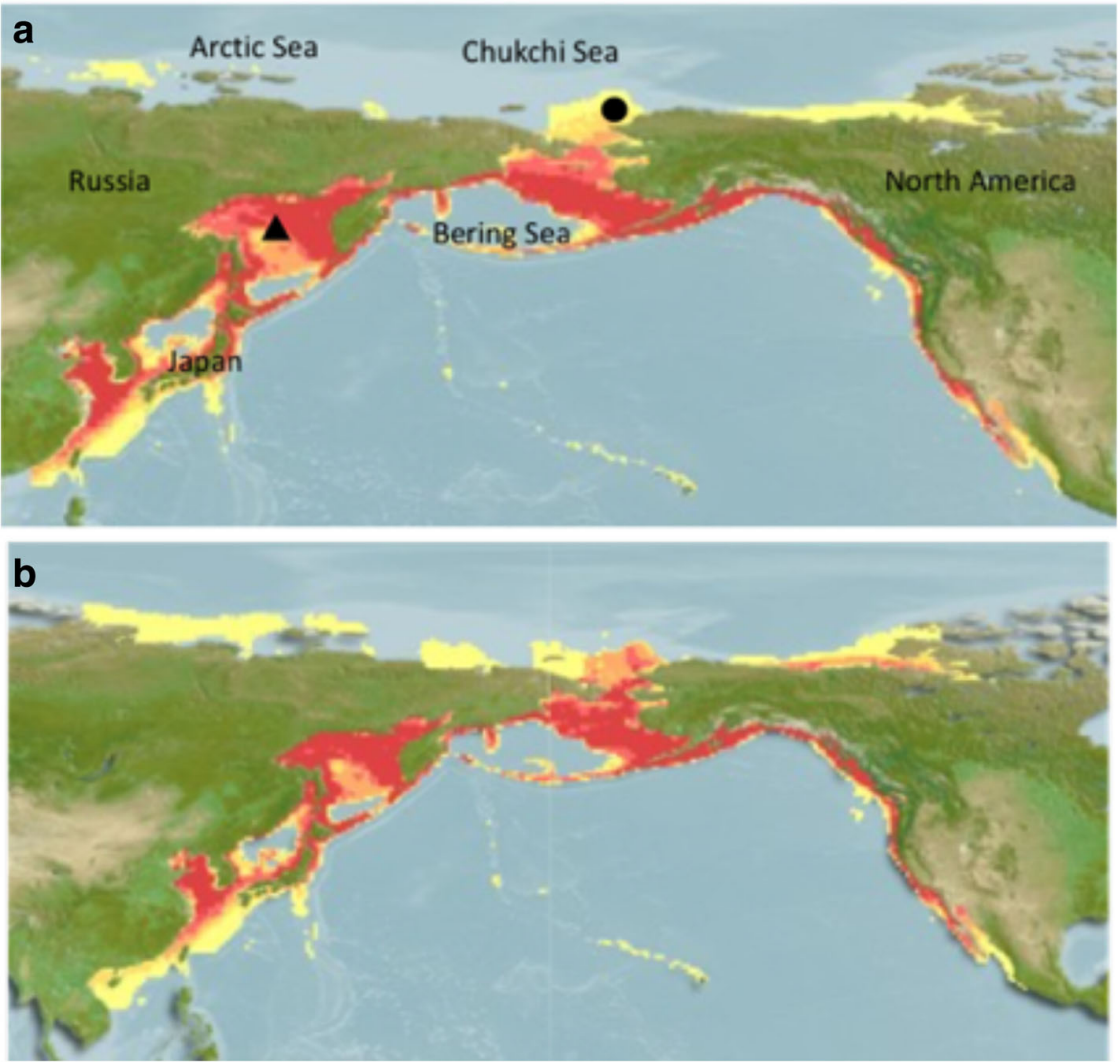
Environmental niche modelling of (**a**) current and (**b**) future (year 2100) suitable habitat for gray whales in the Pacific Ocean. Colours depict the habitat suitability ranging from low (yellow) to high (red). Shapes represent sampling locations for the putative western grey whales (triangle) and eastern grey whales (circle). Feeding grounds are located at higher latitudes, whereas breeding grounds are at lower latitudes

One species severely affected by commercial whaling is the gray whale (Eschrichtius robustus). Gray whales were once common in the Northern hemisphere, but were extirpated from the Atlantic ocean by the early eighteenth century [17], potentially due to environmental change and/or by commercial whaling [2, 18]. Today, gray whales are found in the Eastern Pacific near the coast of North America and the Western Pacific near the coast of Asia (Fig. 1). There is evidence of gene flow between the two “stocks”, but there is also statistically detectable genetic differentiation between them [2, 19]. The eastern gray whale (EGW) population has been extensively studied, and post-whaling estimates based on genetic and ecological data indicate there are ~ 27,000 individuals [19–21]. In contrast, data on the western population is limited [22, 23]. Commercial whaling lasted considerably longer in the western Pacific [24], and today the western gray whale (WGW) is thought to be comprised of < 200 individuals and is listed as ‘critically endangered’ by the International Union for Conservation of Nature (IUCN) [25, 26].

During the late Pleistocene and Holocene (i.e., within the last ~ 100,000 years), the Northern hemisphere experienced massive changes to its marine ecosystems [27]. Glacial periods led to ice cap oscillations that repeatedly opened and closed migration corridors [28–30], and fluctuations in water temperature and sea levels likely forced changes to habitats and feeding modes [2, 3]. Gray whale carrying capacities have been modelled based on shifts in feeding habitat during the last 120,000 years, and those data suggest that multiple demographic bottlenecks may have occurred [3]. In addition to the trophic data, DNA sequences suggest that the EGW population has been subject to a genetic bottleneck during the last century [20]. Although population fluctuations have not been investigated in the WGW, microsatellite and mitochondrial data suggest that the two populations have similar levels of neutral genetic diversity and thus may have similar long-term demographic histories [2, 19].

The ongoing reductions in the extent of sea ice provide gray whales with new potential migration routes and they may be shifting their range farther north in the Arctic [31]. Gray whales have responded to climate changes by shifting the timing of their southbound migration [32]. Because they annually migrate thousands of kilometres from their summer feeding grounds at high latitudes to their winter calving waters at lower latitudes, there may be opportunities for contemporary (i.e., within the last few dozen generations) gene flow between eastern and western Pacific populations (Fig. 1). There are indications of historical (referring to the Pleistocene and early Holocene) gene flow between Atlantic and Pacific gray whales [2], and—more recently—a satellite-tagged WGW has been tracked to an EGW wintering area near the Mexican coast [22]. Furthermore, photographic identification has documented individual gray whales moving between the western Pacific (near Sakhalin Island, Russia) and the eastern Pacific [26]. Collectively, these data suggest that the currently recognized WGW and EGW “populations” of this highly vagile species are not completely independent (i.e., gene flow is possible). Fortunately, population attributes such as historical demography, admixture (i.e., interbreeding between populations that have previously been isolated), and genetic diversity can now be addressed using whole genome sequences [33–35]. The ability to make population inferences from one or a few samples is especially important for rare species, where sampling efforts are often difficult, expensive, and should be minimized because of conservation concerns.

Herein, we employ genomic and computational techniques to infer population attributes of gray whales. The distribution of gray whales is largely disjunct today, but these geographic isolates were demographically and genetically connected in the past (as evidenced by the fact that they are recognized as a single species). Given the recent growth of the EGW population and ongoing climate change, there is reason to suspect that increased gene flow between EGW and WGW may occur in the future. We are interested in the long-term demographic trajectory of gray whales, both from a historical and a future perspective. Given the critically endangered status of the WGW, we were interested in comparing genomes of the WGW and the EGW to investigate levels of genetic diversity as a key component of adaptive potential. We used coalescent-based approaches to retrospectively gauge ancient admixture in gray whale genomes during the Pleistocene, and measures of autozygosity to directly assess inbreeding and search for signals of contemporary differentiation. Our habitat prediction models suggest that novel migratory routes may develop within the next century, which could influence the overall retention of genetic diversity in the species. This study presents the first genomic comparison of gray whales, and extends our insights into the molecular diversity and demographic history of this enigmatic species while contributing to our understanding of how our ocean’s great whales have responded to historical climate change.

## Methods

### Sampling, sequencing and SNP calling

We used previously published whole genome DNA sequences from DeWoody et al. [36]. These sequences were derived from two gray whales sampled near Sakhalin Island, Russia, designated WGW1 (female) and WGW2 (male), and from one putative Eastern gray whale female (EGW) that was beached near Barrow, Alaska (Fig. 1). There is some uncertainty as to true population affinities of these individual gray whales. For example, WGW1 was biopsied near Sakhalin Island in the western Pacific but the same whale has been photographically identified in the eastern Pacific (Laguna San Ignacio; M. Scott, unpublished data). Nevertheless, we assigned geographical names to whales based on sampling locations in order to be comparable with previous genetic work on gray whales [2, 19, 37]. The data utilized herein consisted of 2x100bp paired-end (PE) libraries from each whale (~ 1 billion reads per individual; ~ 700 million high-quality reads per individual after quality-control; Additional file 1: Table S1). For detailed sampling and genome sequencing methodology see DeWoody et al. [36]. For a summary of number of reads per individual and quality control effects, see Additional file 1: Table S1.

We used FASTQC v0.11.2 (www.bioinformatics.babraham.ac.uk/projects/fastqc) to generate summary statistics for the sequencing reads. TRIMMOMATIC v0.32 [38] was used to remove adaptor sequences and trim low quality bases (< 20 Phred scores) from both the 5′ and 3′ end of each read. BWA v0.7.12 [39] was used to map the PE reads to the published genome of the common minke whale (*B. acutorostrata*) (GenBank accession: SAMN02192642, [40]) using the ‘bwtsw’ function that indexes whole genomes, and the ‘mem’ function for mapping. PICARD-TOOLS v2.0.1 (http://broadinstitute.github.io/picard/.) was employed to mark duplicate reads. SAMTOOLS v1.3 [41] was used for alignment manipulation. Local realignment, duplicate removal, and SNP variant calling were carried out with GATK v3.5 [42] following ‘Best Practices protocol’ [43, 44]. Genotypes were called across all three samples together using the ‘gvcf’ option. We used a minimum base quality score of 20 (which corresponds to a base calling error rate of ~ 1% [45]) with a minimum mapping quality score of 20. In the downstream analyses, we only used SNPs with minimum 20× coverage, which should help minimize the number of heterozygotes falsely scored as homozygotes [46, 47]. Eight minke whale scaffolds are X-linked [48], and we removed gray whale reads that mapped to these scaffolds so they would not bias our downstream analyses. None of our gray whale reads mapped to Y-linked scaffolds because [48] reported none in their minke genome assembly.

### Genetic diversity

Nucleotide diversity can be used to assess ancient admixture as well as contemporary differentiation [34, 49, 50]. We estimated observed heterozygosity for each individual, *θ*_genome_, based on the number of heterozygous sites / total number of sites where only sites with minimum 20× coverage were considered. We used *θ* values associated with each individual to independently estimate equilibrium effective population sizes (*N*_*e*_) following *θ* = 4*N*_e_*μ* [51]. To quantify differences in *N*_*e*_ we compared *θ* among individuals, assuming that substitution rates do not vary appreciably across samples.

We directly quantified inbreeding levels by identifying the number and lengths of autosomal runs-of-homozygosity (ROHs) in each individual. A ROH is a genomic region that contains far less nucleotide variation than expected based on the genome–wide average for an individual [52]. Under random mating, the length of ROH regions is expected to decrease with increasing number of generations to the ‘most recent common ancestor’ (MRCA) due to recombination and de novo mutations. In contrast, with inbreeding—as is often the case for critically endangered species—autozygosity is expected to increase over time, thus increasing the number and length of ROHs in the genome each generation. Analysis of ROH abundance and extent thus provides information on a population’s demographic history and on the genetic relationships among individuals [53].

We estimated four different ROH parameters: i) number of ROHs in each genome (*N*_ROH_); ii) the mean length of ROHs (*L*_ROH_); iii) the heterozygosity outside ROHs (*θ*_noROH_); and iv) the inbreeding coefficient *F*_ROH_, the overall proportion of the genome contained in ROHs. We estimated *θ*_noROH_ as the number of heterozygous SNPs / (total number of SNPs – SNPs in ROHs). When *F*_ROH_ is compared to *θ*_genome_, it quantifies the effect of inbreeding on overall levels of genomic variation. To compare our results directly to patterns of ROHs found in other species [54], we used PLINK v1.90b3.36 [55] and defined ROHs as portions of the genome that spanned at least 20 homozygous sites allowing for a single heterozygous SNP (e.g., due to de novo mutation) and 1 missing SNP (e.g., a site with missing data) following Howrigan et al. [56]. We searched the genomes for ROHs in consecutive 20 SNP sliding windows and, to facilitate detecting both short and long ROHs, we set the lower bound for ROHs to 1 kb. We used a Welch two-sample t-test to test for pairwise differences in *L*_ROH_ among individuals whereas pairwise ROH frequency distributions were compared among all three gray whales using a two-sample Kolmogorov–Smirnov test. All statistical tests were conducted in R [57].

### Relatedness and population structure

We used PLINK to measure relatedness among individuals. Pairwise identical-by-state (IBS) comparisons were estimated based on the ratio of probabilities between a heterozygote–heterozygote site, *p*(HetHet) = 4*p*^2^*q*^2^, to the probability of a homozygote–homozygote site, *p*(HomHom) = *p*^*2*^*q*^2^. For each pair of individuals, the number of variable sites where they share no alleles (IBS = 0; e.g., discordant homozygotes AA/BB and BB/AA) are counted along with the number of sites where they share two alleles (IBS = 2) (e.g., heterozygotes AB/AB, BA/BA). On average, we expect this probability ratio to be 1:2 if the pair comes from a randomly mating population [55, 58]. A ‘HetHet’: HomHom’ ratio > 2 suggests that the individuals are more related than expected by chance, and a HetHet’: HomHom’ ratio < 2 suggests that the individuals have recent ancestry from different random mating populations. We used the ‘pairwise population concordance’ (PPC) test to evaluate if this probability ratio significantly deviated from the expected ratio under random mating, applying a significance level of 0.05, a minor allele frequency (MAF) of 0.01, and a minimum distance of 500 k base pairs between informative SNPs to limit the effects of linkage disequilibrium (LD).

### Ancient admixture

To test for ancient admixture, we used the ABBA–BABA *D*-statistic test implemented in ANGSD v0.912 [34, 59]. The *D-*statistic tests for admixture between four individuals: two conspecific individuals (P1 and P2), a potential introgressor (P3), and an outgroup (O). At each polymorphic site in the genome the relationship among these four individuals and the topology of the species tree is compared. Sites that are inconsistent with the species tree are the sites where P2 shares a derived allele with P3 but not P1 (ABBA sites) or P1 shares derived sites with P3 but not P2 (BABA sites). An excess of either ABBA or BABA sites, compared to the sites supporting the species tree (i.e., AABB), is an indication of admixture between P2 and P3 or between P1 and P3, respectively. In the absence of ancient population structure, incomplete lineage sorting is the only process other than admixture that produces inconsistency with the species tree topology, but incomplete lineage sorting is expected to produce ABBA and BABA sites in an equal ratio [49, 50]. The *D*–test statistic evaluates the number (*n*) of ABBA and BABA sites (*D* = (*n*ABBA - *n*BABA) / (*n*ABBA + *n*BABA)) and *D* < 0 means that P1 is more closely related to P3 than to P2, whereas *D* > 0 indicates that P2 is more closely related to P3 than P1. The significance of the *D* test was evaluated with a *Z*-score, where |*Z*-scores| > 3 was used as the critical value for a significant test [50]. As an outgroup, we used the common minke whale. The phylogenetic relationships among baleen whales are not completely resolved [60, 61], but the common minke whale is the closest relative with a published genome sequence [62]. We used an LD block size of 10 Mb; increasing the block size (e.g., 20 Mb, 30 Mb) did not change the outcome of the ABBA-BABA test. We tested all scaffolds > 10 Mb in length in order to obtain a reliable *Z*-score. Admixture *D*-statistics were considered significant for |*Z*-scores| > 3.

### Inference of demographic history

We used the PSMC’ mode implemented in MSMC [33, 63] to infer ancient demographic histories. Eleven scaffolds larger than 30 Mb in length, corresponding to a total of ~ 400 Mb, were used to improve the accuracy of inferring past recombination events [33, 64]. We ran the MSMC analysis for each individual separately using default settings; 20 iterations and averaging over 30 time segments. To quantify the variance in *N*_e_ we bootstrapped using the same MSMC settings. For each individual, 20 bootstrapped datasets were generated by randomly sampling 5 Mb sequences from each of the 11 scaffolds used to trace the mean *N*_e_. Substitution rates—for both mitochondria and nuclear loci—are reportedly 8–10 fold slower in baleen whales than in other mammals [65, 66]. In order to convert *θ* to *N*_e_ over time, we applied an autosomal substitution rate of 4.8 × 10^− 10^ bp^− 1^ year^− 1^ (credibility interval (CI): 1.5 × 10^− 10^ – 10 × 10^− 10^) [67], and a generation time of 18.9 years which corresponds to the midpoint of estimated generation times which range between 15.5 and 22.3 years [68, 69]. MSMC runs were assessed for convergence using the R package CODA [70].

### Prediction of suitable habitat

We used AQUAMAPS [71] to predict the relative probability of the future gray whale distribution across the Northern Hemisphere based on contemporary local conditions. Suitable habitat was based on occurrence records available via Ocean Biogeographic Information System (http://www.iobis.org) using the contemporary environmental envelope settings suggested by Alter et al. [2] (Additional file 1: Table S2), and future (year 2100) envelope settings from AQUAMAPS [72]. We assumed that current environmental conditions are representative of the Holocene, as the Holocene climate has experienced relatively little variation compared to interglacial cycles [73].

## Results

### Genetic diversity

**Fig. 2:**
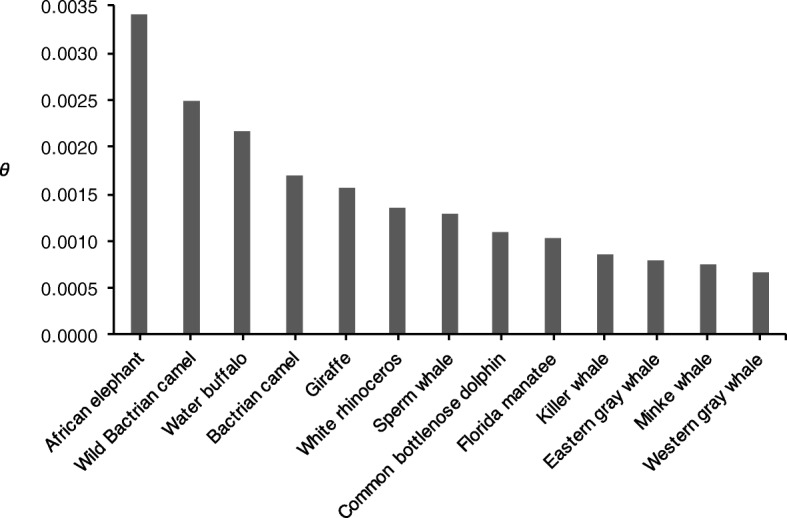
Overview of nuclear genomic diversity (*θ*) in various cetaceans, marine mammals, and large herbivores. Data from the current study and Brüniche-Olsen et al. [54]. Mean *θ* is provided for the two western gray whales in this study

We mapped, from each individual, high-quality PE reads from one eastern and two western gray whales to the minke whale genome. The mean depth of coverage per individual ranged from 27× to 30× (Additional file 1: Table S1), and this relatively deep coverage allowed us to assess nucleotide diversity with confidence. The level of genetic diversity represented by theta (*θ*) was lower in the individuals from the Western population (*θ* = 6.69 × 10^− 4^ and 6.64 × 10^− 4^) relative to the individual from the putative Eastern population *θ* = 8.00 × 10^− 4^ (Fig. 2). Thus, there is about a 1.2-fold difference in genetic diversity between East and West.

### Inbreeding

**Fig. 3:**
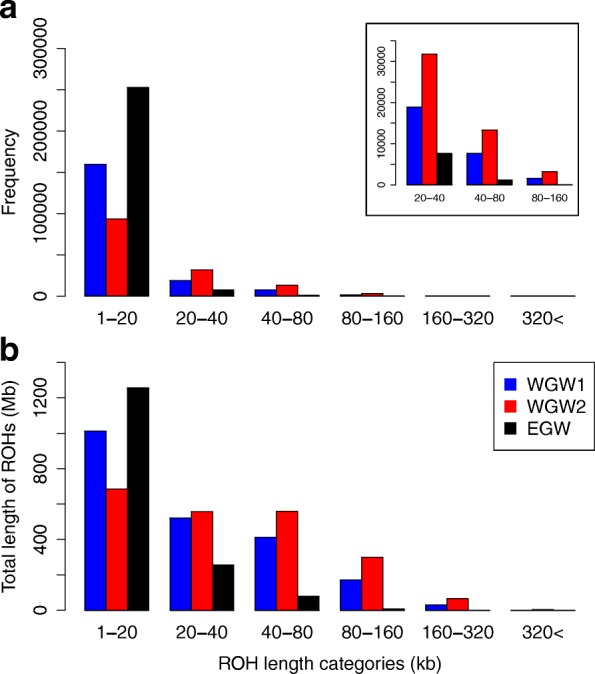
Total number of runs of homozygosity (ROHs) and proportion of ROH size classes in sampled gray whale genomes. Shown for each individual is the number of ROHs in each size class (**a**), with an insert showing the 1-160Kb ROH length categories in detail) and the sum of ROH lengths (Mb) in the genome (**b**). A Kolmogorov–Smirnov test indicated that the pairwise comparisons of ROH frequency distributions between were significantly different from each other (WGW1 & EGW *D* = 0.186, *p =* 2.2 × 10^− 16^; WGW2 & EGW *D* = 0.322, *p =* 2.2 × 10^− 16^, and WGW1 & WGW2 *D* = 0.142 *p =* 2.2 × 10^− 16^), suggesting that there are significant differences in genealogical histories between individuals

We found ROHs ranging from 1 to 559 Kb in length; few were longer than 300Kb (Fig. 3). Estimates of *θ*_genome_ and *θ*_noROH_ were lower in both WGWs than in the EGW (Table 1). The western individuals had fewer ROHs (WGW1: *n*_ROH_ = 188,012 and WGW2: *n*_ROH_ = 126,893) than the Eastern individual (*n*_ROH_ = 263,877), but their mean ROH length were significantly longer (WGW1: *L*_ROH_ = 11Kb and WGW2: *L*_ROH_ = 17Kb; both *p =* 2.2 × 10^− 16^) than in the eastern individual (EGW: *L*_ROH_ = 6Kb). ROHs covered a larger proportion of the western gray whale genomes (WGW1: *T*_ROH_ = 2.1 × 10^6^ bp; WGW2: *T*_ROH_ = 2.2 × 10^6^ bp) compared to the eastern gray whale (EGW: *T*_ROH_ = 1.6 × 10^6^ bp) (Table 1). All individuals differed significantly from one another in *L*_ROH_ (*p =* 2.2 × 10^− 16^), and the Kolmogorov–Smirnov test showed that the ROH distributions were significantly different from one another (WGW1 & EGW *D* = 0.186, *p =* 2.2 × 10^− 16^; WGW2 & EGW *D* = 0.322, *p =* 2.2 × 10^− 16^, and WGW1 & WGW2 *D* = 0.142 *p =* 2.2 × 10^− 16^). This suggest that there are significant differences in genealogical histories between all individuals. Estimates of *F*_ROH_ were 0.088 (WGW1), 0.092 (WGW2), and 0.067 (EGW) and thus on average the WGWs were ~ 1.3 times as inbred as EGW.

**Table 1:** Summary statistics for the gray whales. Heterozygosity across the entire genome (*θ*_genome_), heterozygosity excluding ROHs (*θ*_noROH_), number of ROHs (*N*_ROH_), mean ROH length (*L*_ROH_), sum of ROH lengths (*T*_ROH_), and inbreeding coefficient (*F*_ROH_) in the gray whale autosome. All results are based on sites with depth of coverage ≥20×. A genome size of 2.4Gb was used for calculating *F*_ROH_

Sample	*θ*_genome_	*θ*_noROH_	*N*_ROH_	*L*_ROH_ (×10^3^nt)	*T*_ROH_ (×10^6^nt)	*F*_ROH_
WGW1	6.69 × 10^−4^	6.79 × 10^− 4^	188,012	11.4 (16.0)	2.1	0.088
WGW2	6.64 × 10^−4^	6.74 × 10^− 4^	126,893	17.1 (22.7)	2.2	0.092
EGW	8.00 × 10^−4^	8.11 × 10^− 4^	263,877	6.1 (6.8)	1.6	0.067

### Relatedness and population structure

To evaluate pairwise relatedness, we used the ‘HetHet’ to ‘HomHom’ ratios (where a ratio of 2.0 is expected for individuals from the same random mating population and a ratio > 2.0 suggests that the pair is more related to each other than expected based on chance alone). All pairwise comparisons yielded a ‘HetHet’ to ‘HomHom’ ratio ≥ 2 (Table 2), and the PPC test could not reject the null hypothesis: ‘HetHet’: HomHom’ ratio = 2 (Table 2). Thus this test is uninformative as the three individuals may or may not belong to the same gene pool.

**Table 2:** Relatedness and population clustering. Estimates are based on PLINK genotype calls where the ‘identical by state’ (IBS) genotype pattern was estimated for a pair of samples and the test for population clustering was conducted using pairwise population concordance (PPC). The genotype pattern for each variable site is estimated as the sharing of two ancestral alleles, one ancestral and one derived allele, and two derived alleles between the individuals. The IBS ratios indicate that all pairs (ratios > 2.0) are more related than expected under random mating. The PPC results indicate we cannot reject the null hypothesis (ratio = 2) that all three individuals belong to the same population (*p =* 0.05)

Pair	HomHom	HetHet	Ratio	PPC
WGW1 & WGW2	1533	4377	2.9	1.00
WGW1 & EGW	1384	4513	3.3	1.00
WGW2 & EGW	1572	4252	2.7	1.00

### Ancient admixture

**Fig. 4:**
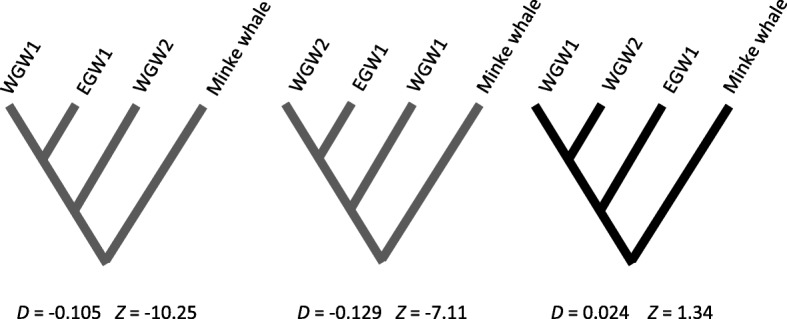
Results from the ABBA-BABA tests for different possible topologies among gray whales from the eastern and western populations when using the common minke whale as the outgroup. The *D-*statistic for each topology is considered statistically significant, meaning the topology can be rejected, if the associated standard score (|*Z*|) has an absolute value > 3. The two gray topologies were both rejected (|*Z*| > 3), but the black topology could not be rejected (|*Z*| < 3). This indicates that the signal of contemporary genomic structure we detected among geographic populations is stronger than the signal of historical admixture. WGW, western gray whale. EGW, eastern gray whale

The ABBA–BABA test revealed no significant support for ancient admixture (e.g., historical panmixia) between the Western and Eastern gray whales (Fig. 4; Additional file 1: Table S3). If ABBA and BABA patterns are equally common, then in theory *D* = 0 and the data are consistent with the tree. Deviations where *D* ≠ 0 can be due to: i) P3 exchanged genes with P1 or P2; ii) ancestral population (P1, P2 and P3’s founder) structure leading to discordant gene trees; or iii) P1 or P2 could have received genes from an unsampled ‘ghost’ population (Pg). The test is not influenced by demographic events assuming that P1, P2 and P3’s ancestral population was panmictic [49], which should be a reasonable assumption for gray whales [2].

### Inference of long-term demographic history

**Fig. 5:**
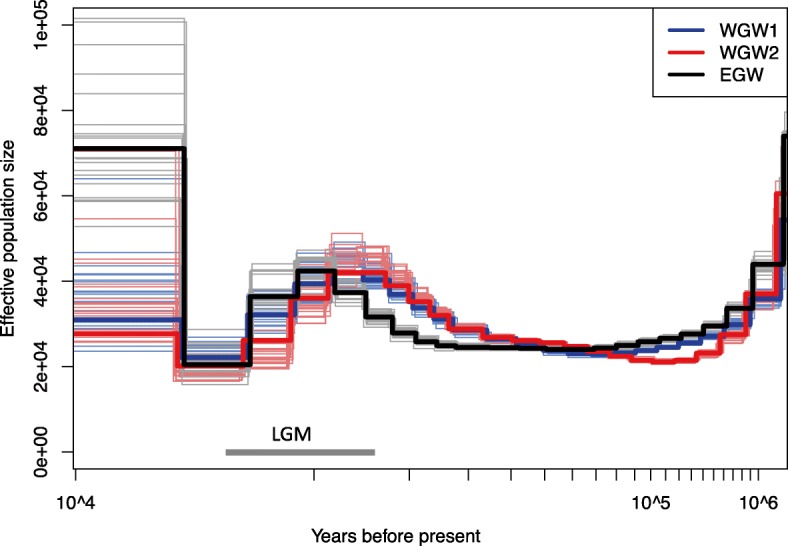
Estimated historical effective population sizes (*N*_e_) for western (red and blue) and eastern (black) gray whales. Thick lines represent the median *N*_e_ and thin light lines of the same colour represent 20 iterations of bootstrap sampling. Estimates represent averages based on 11 autosome scaffolds larger than 30 Mb. An estimated mutation rate of 4.8 × 10^− 10^ bp^− 1^ year^− 1^ and a mean generation time of 18.9 years were used in these PSMC’ analyses. The last glacial maximum (LGM) is indicated with a gray bar

We traced effective population size estimates over the last ~ 1,000,000 years using the PSMC’ method (Fig. 5; Additional file 1: Figure S1). The three individuals exhibit very consistent trajectories, indicating a step decline in *N*_e_ from *N*_e_ > 50,000 in the interval of ~ 1,000,000 years before present (YBP) until 100,000 YBP followed by a more stable period (~ 100,000–30,000 YBP) with *N*_e_ ~ 25,000 for both EGW and WGW populations. Prior to the LGM both populations increase in size to *N*_e_ ~ 45,000; hereafter a reduction in *N*_e_ to a population size of *N*_e_~ 20,000 is observed in all three trajectories. These consistent results among individuals suggest there is relatively little noise in this PSMC’ analysis and that the trajectories themselves are likely a realistic representation of historical population dynamics. Furthermore, the most recent estimate of census population size (*N*_c_) of the EGW is 27,000 [21]. The concordance between *N*_c_ and recent *N*_e_ estimates (Fig. 5) suggests that the substitution rate we used (4.8 × 10^− 10^ bp^− 1^ year^− 1^) is a reasonable approximation of the true genome-wide substitution rate.

### Predictions of suitable habitat

Our environmental niche modelling suggests that current habitat suitability is relatively high from Taiwan to Kamchatka through much of the Bering Sea and along the coast of North America to the Gulf of California (Fig. 1a). Currently marginal habitat, which is expected to improve in the future due to ongoing climate change, includes the Arctic and Chukchi Seas (Fig. 1b).

## Discussion

Anthropogenic factors are rapidly changing the global environment. We think that predictions regarding future biological impacts (e.g., species range shifts) are most informative when presented in a historical context. Genomic data have great potential in this regard as they can be used as a window to the past (e.g., the reconstruction of past demographic histories) and into the future (e.g., by identifying genes expected to face particular selection pressures, such as those related to thermoregulation). Using whole genome data from contemporary eastern and western gray whale populations, we quantified genetic diversity in gray whales and inferred key population attributes that bear on their evolution and conservation.

### Genetic diversity and inbreeding

The genome-wide heterozygosity in gray whales is similar to the minke whale, but lower than other marine mammals—e.g., sperm whales (*Physeter catodon*), common bottlenose dolphin (*Tursiops truncatus*), killer whales (*Orcinus orca*), and manatees (*Trichechus manatus latirostris*)—and considerably lower than terrestrial megafauna (i.e., African elephant (*Loxodonta africana*), camels (*Camelus bactrianus* and *C. ferus*), white rhinoceros (*Ceratotherium simum*)) (Fig. 2). We expect that the variation in *θ* may be explained in part by differences in body size; larger animals have slower mutation rates, longer generation times, and produce fewer offspring—all factors that impact *θ* [74, 75]. Gray whales are the largest of the mammals surveyed here, which could partly explain their low genomic diversity, but population declines over the last ~ 20,000 years (Fig. 5) may also be a significant contributing factor.

Reduced genomic diversity is a concern as it constrains adaptive potential [76]. We observed lower *θ*_genome_ and *θ*_noROH_ in western than eastern gray whales (Table 1), likely due to the smaller size of the western population compared to the eastern population. Small population sizes and reduced gene flow will lead to increased inbreeding that has the potential to reduce reproductive fitness due to homozygosity of deleterious recessive alleles and to reduced heterosis. The extent of ROHs in a genome is correlated with population size reductions and increased consanguinity [52, 53]. Our data indicate that, consistent with contemporary population sizes, ROHs significantly reduce overall nucleotide variation in the gray whale genome (Table 1). The timing and duration of bottlenecks are directly associated with the extent of ROHs; i.e., recent inbreeding leads to long ROHs whereas ancient inbreeding persists in the genome as shorter ROHs that have been disrupted by mutation and recombination [77, 78]. The eastern gray whale had more but shorter ROHs than the western gray whales (Table 1, Fig. 3). This is not surprising given that the eastern population is ~ 100× larger and has not experienced extensive recent inbreeding [20]. In contrast, the western gray whale individuals had fewer but longer ROHs and a larger proportion of their genomes in ROHs (Table 1), a pattern that can be produced by a continuous small population size or a genetic bottleneck that persists for multiple generations [53]. The small size of the western population (< 200 individuals) may not only have led to loss of genetic diversity, but also the loss of adaptive potential in the face of impending environmental change [8–10].

### Relatedness, gene flow and geographical isolates

Gray whales are one of the most vagile species on earth; telemetry and photographic data indicate that some individuals annually move thousands of kilometres across the Pacific [22, 26]. This contemporary movement of individuals between eastern and western populations provides opportunities for gene flow. Furthermore, our niche modelling suggests that gray whales from the east and from the west could encounter the same suitable habitat (Fig. 1b). However, despite the potential overlap in suitable habitat and the known movement of individuals between the populations, their genomes significantly differ in terms of homozygosity (Fig. 3). Thus the ROH data are consistent with previous reports of population structure between eastern and western gray whales [2, 19]. However the PPC test could not reject the null hypothesis of random mating (Table 2; *p =* 1.00) and the relatedness analysis showed that the EGW was more closely related to both of the WGWs than expected by chance. These PPC and relatedness results are consistent with an earlier relatedness analysis based on 88 gene-associated SNPs, which found the EGW was no more or less related to the WGW population than expected on the basis of chance alone [36].

During the Pleistocene, climate-dependent dispersal occurred between the Pacific and Atlantic gray whale populations; prior to the last glacial period (110,000–11,700 YBP) and after the opening of the Bering Strait, gray whales migrated between the Pacific and Atlantic oceans [2]. Analyses of mitochondrial sequences have documented haplotype sharing between the eastern and western populations, suggesting that recent maternal gene flow has occurred during the Holocene [2]. In 2010, a Pacific gray whale was observed in the Mediterranean Sea, a sighting which produced speculation that climate-induced shrinking of the Arctic Sea ice may ultimately enable gray whales to recolonize the Atlantic [79]. Thus, although gene flow between the western and eastern populations has no doubt occurred multiple times since the Pleistocene, the signal of contemporary genomic structure we detected between geographic populations is stronger than the signal of historical admixture (Fig. 4). For all *D*–test statistics we found the two WGWs to be more closely related to each other than either was to the EGW (Additional file 1: Table S3), although we could not reject the hypothesis that the individuals belong to the same randomly mating population (Table 2).

### Dating and severity of population decline (s)

The dating of population size changes is inexact due to errors in the estimation of mutation rates and generation times, but genetic datasets are nevertheless often highly concordant with independent datasets (e.g., fossil evidence; [80, 81]). Demographic histories inferred from single whole genome sequences trace from the two haplotypes to their coalescence in the MRCA. This means that the most recent past is not well resolved, and if unphased haplotypes are used—as done in this study—this also affects deep (past) resolution [63]. Thus, our PSMC’ analyses are unlikely to recover any Anthropocene population size changes associated with commercial whaling, as any genetic signal this may have left is much too recent for this method to detect. That said, trajectories of *N*_e_ over the last ~ 1,000,000 years are highly consistent with one another and suggest a similar demographic history in each lineage (Fig. 5). Pre-whaling eastern gray whale census population size (*N*_c_) has been estimated at 96,000 (CI: 76,000–118,000) individuals based on nuclear microsatellites [67], whereas mitochondrial DNA sequences [20] yield *N*_c_ estimates of 100,670 (90% HPD: 59,940–111,550). These *N*_c_ estimates correspond to *N*_e_ of ~ 32,000 (CI: 25,000–39,000) for microsatellites, and *N*_e_ ~ 17,000 (90% HPD: 10,000–19,000) for mitochondrial DNA, which is similar to our post LGM *N*_e_ estimate ~ 20,000 (Fig. 5). These differences among studies may illustrate that using a subset of genomic markers does not accurately capture overall genomic diversity perhaps because of the ascertainment bias associated with the selection of highly polymorphic markers such as microsatellites [82].

Our data suggest an ancient population decline during previous ice ages and a more recent decline in the last ~ 25,000 years (Fig. 5). Glacial periods are often associated with population declines, and the large shifts in climate have impacted both terrestrial [83, 84] and marine mammals [85–88]. Taken together with the evidence for contemporary bottlenecks—occurring around the time of commercial whaling [20]—these results support population models which indicate multiple bottlenecks have occurred in gray whales [3]. Cumulatively, these bottlenecks may have contributed to the relative paucity of genetic diversity observed in gray whales (Fig. 2).

### Western gray whales (WGWs)

We were particularly interested in tracing the demographic history and quantifying genetic diversity within the WGW because of its conservation status. We found that WGWs had increased autozygosity (higher *F*_ROH_) and lower *θ*_genome_ (Table 2) compared to the eastern gray whale, both of which would be expected in a small inbred population [52]. However, despite having a more than 100–fold difference in census population size, the genomic differences were modest as *N*_e_ only differed 1.2 fold between the two geographic populations. The observed ROH patterns suggest that the western population has experienced population size reduction and an elevated level of inbreeding relative to the eastern individual (Fig. 3). These ROH patterns likely result from recent processes (e.g., inbreeding and drift) as opposed to a long-term small population size, which should be reflected in the Pleistocene *N*_e_ (Fig. 5). The small population size and low genetic diversity limit the potential evolutionary responses to future environmental change, and thus ongoing efforts to conserve the WGW are critical. Our samples sizes are large in terms of number of genetic loci, but small in terms of individual animals. Future studies will reveal whether the patterns we observe herein are indicative of the species as a whole.

## Conclusion

Whole genome sequencing of cetaceans provides new insights into how these enigmatic animals have responded to past and ongoing changes in the marine environment. Herein, we present the first genome-scale study of gray whale demographic history. Our results show that gray whales from the eastern and western Pacific have low genetic diversity, that the past gray whale population (s) was much larger and experienced multiple declines since the Pleistocene, and that there is some evidence of geographic structuring between the populations. Ecological predictions for the year 2100 suggest the current habitat of gray whales in the Pacific Ocean is unlikely to decrease while their former habitat in the Atlantic Ocean could expand with global warming [2]. Combined with decreasing sea ice cover in the Arctic, this expanding habitat could provide gray whales with opportunities to use alternative migration routes that could genetically bind east and west [31] but only time will tell how anthropogenic effects, genetic drift, inbreeding, and climate change will impact the population viability of gray whales over the long-term.

## Additional file

Additional file 1: **Table S1.** Information on raw reads filtering statistics. Paired-end libraries were sequenced on an Illumina HiSeq 2500. **Table S2.** Environmental variables used in AQUAMAPS to generate maps of suitable habitat for gray whales during the Holocene. **Table S3.** The *D*–test statistic evaluates the number (*n*) of ABBA and BABA sites (*D* = (*n*ABBA - *n*BABA) / (*n*ABBA + *n*BABA)) and *D* < 0 means that P1 is more closely related to P3 than to P2, whereas *D* > 0 indicates that P2 is more closely related to P3 than P1. The significance of the *D* test was evaluated with a *Z*-score, where |*Z*-scores| > 3 was used as the critical value for a significant test. **Figure S1.** Inferred effective population sizes (*N*_e_) over time. Estimates are averages based on 11 autosomal scaffolds larger than 30 Mb. A substitution rate of a) 10 × 10^− 10^ bp^− 1^ year^− 1^ and b) 1.5 × 10^− 10^ bp^− 1^ year^− 1^ were used. (DOCX 446 kb)

## Abbreviations

CI: credibility interval
EGW: eastern gray whale
*F*_ROH_: inbreeding coefficient
IBS: identical-by-state
IUCN: International Union for Conservation of Nature
IWC: International Whaling Commission
LD: linkage disequilibrium
LGM: Last Glacial Maximum
MAF: mean length of ROHs (*L*_ROH_) minor allele frequency
MRCA: most recent common ancestor
*N*_e_: effective population sizes
*N*_ROH_: number of ROHs
O: outgroup
P1; P2: conspecific individuals
P3: potential introgressor
PPC: Pairwise population concordance test
ROH: runs-of-homozygosity
WGW: western gray whale
YBP: years before present
*θ*: theta
*θ*_genome_: observed heterozygosity
*θ*_noROH_: heterozygosity outside ROHs
