# Supplementary material for: The inference of gray whale (Eschrichtius robustus) historical population attributes from whole-genome sequences
Source: BMC Evol Biol. 2018 Jun 7;18:87. doi: 10.1186/s12862-018-1204-3 (PMC5992727; doi:10.1186/s12862-018-1204-3)
Supplement: Supplementary file 1 — Table S1. Information on raw reads filtering statistics. Paired-end libraries were sequenced on an Illumina HiSeq 2500. Table S2. Environmental variables used in AQUAMAPS to generate maps of suitable habitat for gray whales during the Holocene. Table S3. The D–test statistic evaluates the number (n) of ABBA and BABA sites (D = (nABBA - nBABA) / (nABBA + nBABA)) and D < 0 means that P1 is more closely related to P3 than to P2, whereas D > 0 indicates that P2 is more closely related to P3 than P1. The significance of the D test was evaluated with a Z-score, where |Z-scores| > 3 was used as the critical value for a significant test. Figure S1. Inferred effective population sizes (Ne) over time. Estimates are averages based on 11 autosomal scaffolds larger than 30 Mb. A substitution rate of a) 10 × 10− 10 bp− 1 year− 1 and b) 1.5 × 10− 10 bp− 1 year− 1 were used. (DOCX 446 kb) [file 12862_2018_1204_MOESM1_ESM.docx]

**Supplementary material for “The inference of gray whale population attributes from whole-genome sequences”**

**Table S1.** Information on raw reads filtering statistics. Paired-end libraries were sequenced on an Illumina HiSeq 2500.

| **Sample** | **Insert size (bp)** | **Number of raw read pairs** | **Total number of bases (Gb)** | **% filtered reads after trimming and adaptor removal** | **Estimated sequencing depth (X) from proper read pairs*** |
| --- | --- | --- | --- | --- | --- |
| WGW1 | 300 | 988,439,854 | 97.1 | 75% | 26.5 |
| WGW2 | 300 | 974,126,824 | 95.9 | 78% | 27.1 |
| EGW1 | 300 | 893,146,780 | 88.3 | 95% | 30.3 |

* We assumed that the gray whale genome is similar in size to other Baleen whales (~2.8GB).

**Table S2.** Environmental variables used in AQUAMAPS to generate maps of suitable habitat for gray whales during the Holocene.

|  | **Min.** | **Preferred min. (10^th^ percentile)** | **Preferred max. (90^th^ percentile)** | **Max.** |
| --- | --- | --- | --- | --- |
| Depth (m) | 1 | 30 | 500 | 2 500 |
| Temperature (ºC) | -2 | 3 | 19 | 30 |
| Salinity (PSU) | 20.00 | 31.21 | 33.70 | 35.34 |
| Sea ice concentration | 0.00 | 0.00 | 0.13 | 0.90 |

**Table S3.**  D-statistics admixture analysis. The *D*–test statistics evaluates the number (*n*) of ABBA and BABA sites (*D* = (*n*ABBA - *n*BABA)/(*n*ABBA + *n*BABA)) and *D*<0 means that P1 is closer related to P3 than P2, and *D*>0 that P2 is closer related to P3 than P1.

| **P1** | **P2** | **P3** | **nABBA** | **nBABA** | **Dstat** | **jackEst** | **SE** | **\|Z\|** |
| --- | --- | --- | --- | --- | --- | --- | --- | --- |
| WGW2 | EGW1 | WGW1 | 9096 | 11801 | -0.129 | -0.129 | 0.018 | 7.11 |
| WGW1 | EGW1 | WGW2 | 9551 | 11801 | -0.105 | -0.105 | 0.010 | 10.25 |
| WGW1 | WGW2 | EGW1 | 9551 | 9096 | 0.024 | 0.024 | 0.018 | 1.34 |

**Table S4.** Numts found in the gray whale genome. % identity refers to the identity between the mtDNA and presumptive nuclear DNA sequences.

| **Scaffold #** | **Scaffold**  **length** | **Scaffold**  **start** | **Scaffold**  **end** | **Alignment**  **length** | **mtDNA**  **start** | **mtDNA**  **end** | **% identity** | **# gaps** | **# mismatches** |
| --- | --- | --- | --- | --- | --- | --- | --- | --- | --- |
| 2934009 | 309038 | 15689 | 12713 | 3004 | 7019 | 10010 | 79.0 | 39 | 593 |
|  |  | 16615 | 15740 | 892 | 6168 | 7058 | 82.0 | 17 | 144 |
| 2956425 | 293020 | 157923 | 164839 | 7011 | 1 | 6973 | 81.9 | 132 | 1135 |
| 2953476 | 257138 | 232857 | 231726 | 1141 | 1701 | 2835 | 86.2 | 15 | 142 |
|  |  | 235578 | 235427 | 152 | 657 | 808 | 90.1 | 0 | 15 |
|  |  | 244366 | 235925 | 8552 | 6961 | 15486 | 78.6 | 136 | 1692 |
| 2937584 | 245080 | 9229 | 7536 | 1701 | 5150 | 6825 | 80.9 | 32 | 293 |
|  |  | 14426 | 9353 | 5218 | 1 | 5163 | 79.9 | 199 | 850 |
| 2945058 | 234793 | 9279 | 7950 | 1396 | 2124 | 3507 | 75.0 | 78 | 271 |
| 2941083 | 226373 | 118185 | 117867 | 329 | 15806 | 16132 | 82.1 | 12 | 47 |
| 2941606 | 188681 | 77685 | 77448 | 241 | 2276 | 2515 | 91.7 | 4 | 16 |
| 2961081 | 183993 | 152574 | 147504 | 5106 | 9710 | 14766 | 78.3 | 84 | 1025 |
| 2945783 | 141754 | 48139 | 50023 | 1904 | 10178 | 12066 | 78.3 | 34 | 379 |
|  |  | 52368 | 52566 | 205 | 14039 | 14241 | 87.8 | 8 | 17 |
|  |  | 52829 | 54038 | 1216 | 14275 | 15486 | 81.3 | 10 | 218 |
| 2949731 | 128115 | 39054 | 37395 | 1668 | 5286 | 6947 | 84.5 | 14 | 245 |
|  |  | 39967 | 39054 | 920 | 4306 | 5225 | 84.2 | 6 | 139 |
| 2962175 | 122267 | 20192 | 20470 | 279 | 9098 | 9376 | 84.2 | 0 | 44 |
| 2933531 | 122071 | 2 | 1701 | 1752 | 628 | 2337 | 80.7 | 94 | 244 |
|  |  | 1993 | 4075 | 2120 | 2348 | 4440 | 79.4 | 64 | 372 |
| 2951364 | 119652 | 28604 | 35443 | 6924 | 9321 | 16220 | 80.6 | 108 | 1238 |
|  |  | 35660 | 36343 | 692 | 36 | 723 | 86.4 | 12 | 82 |
| 2940321 | 108448 | 100067 | 97737 | 2363 | 12347 | 14695 | 78.6 | 46 | 459 |
| 2955385 | 97851 | 85688 | 81449 | 4270 | 1 | 4241 | 86.5 | 59 | 519 |
|  |  | 90578 | 86415 | 4193 | 9755 | 13938 | 81.3 | 38 | 748 |
|  |  | 95562 | 90578 | 5049 | 4685 | 9713 | 82.1 | 84 | 821 |
| 2948758 | 90923 | 83430 | 89657 | 6256 | 4027 | 10253 | 80.2 | 57 | 1183 |
| 2962547 | 90767 | 82288 | 83996 | 1712 | 11321 | 13030 | 79.7 | 5 | 342 |
| 2956446 | 83548 | 16401 | 18527 | 2137 | 8349 | 10474 | 79.6 | 21 | 415 |
| 2940828 | 82503 | 14214 | 12763 | 1453 | 3407 | 4859 | 93.0 | 1 | 101 |
|  |  | 14215 | 15502 | 1288 | 12872 | 14159 | 93.2 | 0 | 87 |
|  |  | 15551 | 15509 | 43 | 4810 | 4852 | 93.0 | 0 | 3 |
| 2929206 | 82281 | 11203 | 11541 | 348 | 9203 | 9549 | 82.2 | 10 | 52 |
|  |  | 11551 | 17587 | 6084 | 9526 | 15579 | 81.8 | 77 | 1029 |
| 2959257 | 78689 | 36174 | 38010 | 1856 | 1625 | 3473 | 85.5 | 26 | 244 |
|  |  | 38011 | 39074 | 1070 | 14418 | 15481 | 81.0 | 12 | 191 |
|  |  | 40000 | 41491 | 1533 | 1 | 1524 | 82.5 | 50 | 219 |
| 2945354 | 78626 | 77939 | 77555 | 385 | 3805 | 4189 | 83.4 | 0 | 64 |
|  |  | 78626 | 77980 | 648 | 3122 | 3763 | 84.7 | 7 | 92 |
| 2941710 | 75070 | 11980 | 12774 | 811 | 95 | 900 | 82.0 | 21 | 125 |
|  |  | 13406 | 14079 | 700 | 885 | 1574 | 76.7 | 36 | 127 |
|  |  | 14431 | 14961 | 545 | 1591 | 2131 | 82.6 | 18 | 77 |
|  |  | 15085 | 15781 | 713 | 2122 | 2828 | 82.7 | 22 | 101 |
| 2942428 | 71897 | 44429 | 43565 | 866 | 4243 | 5106 | 80.7 | 3 | 164 |
|  |  | 48590 | 44429 | 4237 | 1 | 4213 | 82.9 | 99 | 627 |
|  |  | 50437 | 48914 | 1586 | 14555 | 16132 | 77.7 | 70 | 284 |
| 2953684 | 68702 | 59361 | 61297 | 1988 | 187 | 2147 | 79.3 | 78 | 334 |
| 2943785 | 67053 | 6227 | 8266 | 2065 | 1008 | 3062 | 87.1 | 35 | 231 |
|  |  | 8317 | 10741 | 2452 | 3121 | 5566 | 84.6 | 33 | 344 |
|  |  | 10744 | 17939 | 7221 | 5728 | 12928 | 82.1 | 45 | 1249 |
|  |  | 19087 | 19697 | 613 | 14154 | 14764 | 84.7 | 4 | 90 |
| 2934590 | 55958 | 51535 | 49686 | 1867 | 7884 | 9749 | 79.9 | 18 | 358 |
| 2953681 | 54612 | 33870 | 31930 | 1978 | 1732 | 3680 | 77.9 | 66 | 372 |
|  |  | 35760 | 34627 | 1202 | 341 | 1520 | 76.3 | 90 | 195 |
| 2960797 | 53815 | 37556 | 34659 | 2925 | 749 | 3661 | 86.8 | 39 | 346 |
|  |  | 38042 | 37822 | 221 | 545 | 764 | 84.6 | 1 | 33 |
|  |  | 47086 | 46822 | 272 | 1 | 271 | 83.8 | 8 | 36 |
|  |  | 49424 | 47119 | 2346 | 14060 | 16380 | 79.8 | 65 | 408 |
| 2958828 | 51990 | 19594 | 15399 | 4245 | 990 | 5216 | 82.0 | 67 | 696 |
| 2956193 | 49569 | 20431 | 14525 | 5972 | 1 | 5948 | 86.0 | 89 | 749 |
|  |  | 30672 | 20464 | 10292 | 6133 | 16380 | 81.4 | 127 | 1787 |
| 2930300 | 49209 | 27730 | 28315 | 598 | 927 | 1522 | 82.4 | 14 | 91 |
|  |  | 28327 | 31680 | 3385 | 1567 | 4908 | 82.6 | 74 | 515 |
| 2934467 | 46240 | 2845 | 4039 | 1232 | 1 | 1218 | 79.1 | 51 | 207 |
|  |  | 4763 | 5406 | 654 | 1225 | 1873 | 80.7 | 15 | 111 |
|  |  | 5406 | 6520 | 1132 | 1840 | 2962 | 83.0 | 26 | 166 |
|  |  | 6851 | 8519 | 1698 | 3020 | 4702 | 78.4 | 44 | 322 |
| 2960162 | 43823 | 13947 | 12125 | 1867 | 81 | 1926 | 81.1 | 65 | 288 |
| 2930949 | 41254 | 14939 | 12255 | 2722 | 3142 | 5845 | 80.3 | 55 | 480 |
| 2933275 | 40245 | 26281 | 21108 | 5251 | 10972 | 16199 | 78.6 | 100 | 1023 |
| 2956499 | 35092 | 12267 | 4516 | 7888 | 3908 | 11744 | 76.8 | 187 | 1642 |
|  |  | 14064 | 12269 | 1830 | 1815 | 3632 | 80.8 | 46 | 305 |
| 2940917 | 33370 | 3427 | 10120 | 6779 | 8735 | 15481 | 78.5 | 117 | 1340 |
| 2937170 | 25761 | 16016 | 21929 | 6067 | 7783 | 13807 | 75.4 | 195 | 1299 |
| 2936112 | 23545 | 10837 | 11530 | 700 | 4530 | 5227 | 81.1 | 8 | 124 |
|  |  | 11555 | 18338 | 6858 | 8915 | 15745 | 78.2 | 101 | 1395 |
|  |  | 18978 | 22808 | 3894 | 1 | 3864 | 83.3 | 93 | 557 |
| 2934812 | 20322 | 9744 | 10952 | 1236 | 7052 | 8277 | 76.3 | 37 | 256 |
|  |  | 14409 | 19004 | 4670 | 10831 | 15480 | 77.2 | 94 | 972 |
| 2951618 | 16620 | 9739 | 12555 | 2860 | 13545 | 16389 | 80.6 | 58 | 498 |
|  |  | 12577 | 15580 | 3037 | 1 | 3025 | 87.0 | 45 | 350 |
| 2949396 | 16323 | 13863 | 13093 | 784 | 1 | 780 | 87.8 | 17 | 79 |
| 2935379 | 15953 | 1344 | 630 | 723 | 1 | 721 | 83.1 | 10 | 112 |
|  |  | 3389 | 2355 | 1043 | 14064 | 15105 | 82.5 | 9 | 174 |
| 2945574 | 15892 | 3465 | 1072 | 2473 | 1 | 2455 | 81.6 | 97 | 357 |
| 2946704 | 14517 | 7430 | 8357 | 951 | 1 | 941 | 79.8 | 33 | 159 |
| 2874705 | 14318 | 10249 | 10 | 10377 | 1 | 10342 | 82.3 | 172 | 1668 |
| 2926406 | 12410 | 3939 | 8309 | 4445 | 11714 | 16132 | 78.3 | 100 | 865 |
| 2937294 | 11395 | 6305 | 1 | 6354 | 1 | 6332 | 85.7 | 71 | 837 |
|  |  | 9579 | 6344 | 3305 | 13098 | 16375 | 79.4 | 96 | 585 |
| 2943801 | 10613 | 7292 | 7555 | 278 | 15857 | 16132 | 79.1 | 16 | 42 |
|  |  | 8008 | 9261 | 1302 | 1 | 1290 | 80.3 | 60 | 197 |
|  |  | 9272 | 10612 | 1365 | 1272 | 2625 | 84.4 | 35 | 178 |
| 2790686 | 9454 | 3571 | 7734 | 4300 | 1 | 4264 | 80.5 | 172 | 666 |
| 2931956 | 7844 | 1706 | 66 | 1663 | 4986 | 6635 | 75.8 | 35 | 367 |
| 2949850 | 6700 | 3422 | 1333 | 2092 | 4806 | 6896 | 86.3 | 3 | 284 |
|  |  | 4764 | 3748 | 1021 | 3805 | 4821 | 82.5 | 8 | 171 |
|  |  | 6259 | 4805 | 1458 | 2314 | 3763 | 85.5 | 11 | 200 |
| 2927476 | 6446 | 3274 | 5868 | 2686 | 1 | 2651 | 77.9 | 126 | 467 |
| 2951985 | 6255 | 1212 | 5543 | 4411 | 11850 | 16219 | 79.5 | 120 | 783 |
|  |  | 5731 | 6253 | 533 | 1 | 530 | 85.6 | 13 | 64 |
| 2745127 | 5965 | 3 | 2368 | 2426 | 2311 | 4674 | 77.2 | 122 | 431 |
| 2728038 | 4136 | 9 | 2510 | 2509 | 9173 | 11673 | 82.2 | 15 | 432 |
|  |  | 2522 | 2954 | 437 | 3546 | 3980 | 86.7 | 6 | 52 |
| 2936218 | 3379 | 3277 | 1894 | 1405 | 4706 | 6086 | 74.2 | 45 | 318 |
| 2754772 | 738 | 112 | 738 | 628 | 2554 | 3180 | 85.4 | 2 | 90 |
| 2884685 | 455 | 21 | 303 | 283 | 10326 | 10608 | 80.6 | 0 | 55 |

*Numts*

Nuclear copies of mtDNA, or "numts", are cytoplasmic mtDNA fragments that have integrated into the host nuclear genome (Lopez *et al.* 1994; Richly & Leister 2004). Numts have the potential to bias evolutionary analyses if mistaken for mitochondrial genes, but also provide valuable genetic markers (e.g., for phylogenetic inference (Triant & DeWoody 2007, 2009)). Numts have been found in most mammals, including cetaceans (Ko *et al.* 2015). We attempted to catalogue gray whale numts to distinguish them from true mtDNA sequences. We did so by BLASTing a gray whale mitochondrial reference genome (Genbank accession: AJ554053) against a draft assembly of the complete gray whale genome (DeWoody *et al.* 2017). We then compared our catalogue of numts to published mtDNA haplotypes (Genbank accession: KJ865243.1 and KJ865244.1) in an effort to determine if rare or divergent haplotypes (e.g., lineage B which is found in WGWs (Meschersky *et al.* 2015)) might mistakenly represent numts as opposed to mtDNA.

**Table S5.** Number of pairwise differences (above the diagonal) and % identity (below the diagonal) among the reference *cyt*B mtDNA sequence (1,137bp) from an eastern gray whale (Genbank accession: AJ554053) and mtDNA lineages A (Genbank accession: KJ865243.1) and B (Genbank accession: KJ865244.1) from western gray whales.

|  | **Lineage A** | **Lineage B** | **Reference** |
| --- | --- | --- | --- |
| **Lineage A** | — | 9 | 3 |
| **Lineage B** | 99.21 | — | 8 |
| **Reference** | 99.74 | 99.30 | — |

**Table S6.** *Cyt*B sequences from a reference (eastern gray whale) and two western gray whale cytb lineages (A and B).

| **Lineage** | **Scaffold #** | **Scaffold**  **length** | **Scaffold**  **start** | **Scaffold**  **end** | **Alignment**  **length** | **mtDNA**  **start** | **mtDNA**  **end** | **% identity** | **# gaps** | **# mismatches** |
| --- | --- | --- | --- | --- | --- | --- | --- | --- | --- | --- |
| lineageA | 2926406 | 12410 | 6424 | 7549 | 1131 | 14 | 1137 | 80.5 | 12 | 208 |
| lineageB | 2926406 | 12410 | 6424 | 7549 | 1128 | 14 | 1137 | 80.8 | 6 | 211 |
| reference | 2926406 | 12410 | 6424 | 7549 | 1128 | 14 | 1137 | 80.4 | 6 | 215 |
| lineageA | 2929206 | 82281 | 16200 | 17348 | 1155 | 1 | 1137 | 81.7 | 24 | 187 |
| lineageB | 2929206 | 82281 | 16200 | 17348 | 1155 | 1 | 1137 | 82.2 | 24 | 182 |
| reference | 2929206 | 82281 | 16200 | 17348 | 1155 | 1 | 1137 | 81.6 | 24 | 188 |
| lineageA | 2934812 | 20322 | 17732 | 18830 | 1112 | 1 | 1109 | 79.4 | 16 | 213 |
| lineageB | 2934812 | 20322 | 17732 | 18830 | 1112 | 1 | 1109 | 79.3 | 16 | 214 |
| reference | 2934812 | 20322 | 17732 | 18830 | 1112 | 1 | 1109 | 79.3 | 16 | 214 |
| lineageA | 2937294 | 11395 | 8495 | 7365 | 1138 | 3 | 1137 | 82.5 | 10 | 189 |
| lineageB | 2937294 | 11395 | 8495 | 7365 | 1138 | 3 | 1137 | 82.6 | 10 | 188 |
| reference | 2937294 | 11395 | 8495 | 7365 | 1138 | 3 | 1137 | 82.4 | 10 | 190 |
| lineageA | 2940917 | 33370 | 8844 | 9977 | 1140 | 1 | 1137 | 80.0 | 9 | 219 |
| lineageB | 2940917 | 33370 | 8844 | 9977 | 1138 | 1 | 1137 | 80.1 | 5 | 222 |
| reference | 2940917 | 33370 | 8844 | 9977 | 1138 | 1 | 1137 | 79.9 | 5 | 224 |
| lineageA | 2942428 | 71897 | 50437 | 49704 | 750 | 355 | 1104 | 81.5 | 16 | 123 |
| lineageB | 2942428 | 71897 | 50437 | 49704 | 750 | 355 | 1104 | 81.7 | 16 | 121 |
| reference | 2942428 | 71897 | 50437 | 49704 | 750 | 355 | 1104 | 81.3 | 16 | 124 |
| lineageA | 2943785 | 67053 | 19134 | 19688 | 555 | 1 | 555 | 83.8 | 0 | 90 |
| lineageB | 2943785 | 67053 | 19134 | 19688 | 555 | 1 | 555 | 83.8 | 0 | 90 |
| reference | 2943785 | 67053 | 19134 | 19697 | 566 | 1 | 564 | 83.6 | 4 | 89 |
| lineageA | 2951618 | 16620 | 10386 | 11521 | 1138 | 1 | 1137 | 82.6 | 3 | 195 |
| lineageB | 2951618 | 16620 | 10386 | 11521 | 1138 | 1 | 1137 | 82.9 | 3 | 192 |
| reference | 2951618 | 16620 | 10386 | 11521 | 1138 | 1 | 1137 | 82.5 | 3 | 196 |
| lineageA | 2953476 | 257138 | 237204 | 236086 | 1129 | 1 | 1124 | 81.5 | 15 | 194 |
| lineageB | 2953476 | 257138 | 237204 | 236086 | 1126 | 1 | 1124 | 81.7 | 9 | 197 |
| reference | 2953476 | 257138 | 237204 | 236086 | 1126 | 1 | 1124 | 81.4 | 9 | 201 |
| lineageA | 2956193 | 49569 | 22626 | 21494 | 1141 | 1 | 1137 | 81.9 | 12 | 195 |
| lineageB | 2956193 | 49569 | 22626 | 21494 | 1141 | 1 | 1137 | 82.1 | 12 | 192 |
| reference | 2956193 | 49569 | 22626 | 21494 | 1141 | 1 | 1137 | 81.8 | 12 | 196 |
| lineageA | 2959257 | 78689 | 38011 | 38930 | 925 | 218 | 1137 | 80.6 | 10 | 169 |
| lineageB | 2959257 | 78689 | 38011 | 38930 | 925 | 218 | 1137 | 80.8 | 10 | 168 |
| lineageA | 2926406 | 12410 | 6424 | 7549 | 1131 | 14 | 1137 | 80.5 | 12 | 208 |
| lineageB | 2926406 | 12410 | 6424 | 7549 | 1128 | 14 | 1137 | 80.8 | 6 | 211 |
| reference | 2926406 | 12410 | 6424 | 7549 | 1128 | 14 | 1137 | 80.4 | 6 | 215 |
| lineageA | 2929206 | 82281 | 16200 | 17348 | 1155 | 1 | 1137 | 81.7 | 24 | 187 |
| lineageB | 2929206 | 82281 | 16200 | 17348 | 1155 | 1 | 1137 | 82.2 | 24 | 182 |

**a)**

**b)**

**Figure S1.** Inferred effective population sizes (*N*_e_) over time. Estimates are averages based on 11 autosomal scaffolds larger than 30Mb. A substitution rate of a) 10×10^-10^ bp^-1^ year^-1^ and b) 1.5×10^-10^ bp^-1^ year^-1^ were used.

**References.**

DeWoody JA, Fernandez NB, Brüniche-Olsen A*, et al.* (2017) Characterization of the Gray Whale Eschrichtius robustus Genome and a Genotyping Array Based on Single-Nucleotide Polymorphisms in Candidate Genes. *The Biological Bulletin* **0**, 000-000.

Ko Y-J, Yang EC, Lee J-H*, et al.* (2015) Characterization of cetacean Numt and its application into cetacean phylogeny. *Genes & Genomics* **37**, 1061-1071.

Lopez JV, Yuhki N, Masuda R, Modi W, O'Brien SJ (1994) Numt, a recent transfer and tandem amplification of mitochondrial DNA to the nuclear genome of the domestic cat. *Journal of Molecular Evolution* **39**, 174-190.

Meschersky IG, Kuleshova MA, Litovka DI*, et al.* (2015) Occurrence and distribution of mitochondrial lineages of gray whales (Eschrichtius robustus) in Russian Far Eastern seas. *Biology Bulletin* **42**, 34-42.

Richly E, Leister D (2004) NUMTs in Sequenced Eukaryotic Genomes. *Molecular Biology and Evolution* **21**, 1081-1084.

Triant DA, DeWoody JA (2007) The Occurrence, Detection, and Avoidance of Mitochondrial DNA Translocations in Mammalian Systematics and Phylogeography. *Journal of Mammalogy* **88**, 908-920.

Triant DA, DeWoody JA (2009) Demography and Phylogenetic Utility of Numt Pseudogenes in the Southern Red-Backed Vole (*Myodes gapperi*). *Journal of Mammalogy* **90**, 561-570.
